# Integrating molecular dynamics simulations to enable rational assembly of immune signals for immunotherapy

**DOI:** 10.1039/d6nh00215c

**Published:** 2026-05-30

**Authors:** Eugene Froimchuk, Meenal Jain, Abhilash Sahoo, Camilla Edwards, Shrey Shah, Robert S. Oakes, Silvina Matysiak, Christopher M. Jewell

**Affiliations:** a Robert E. Fischell Institute for Biomedical Devices College Park MD USA cmjewell@umd.edu https://www.jewell.umd.edu; b Fischell Department of Bioengineering, University of Maryland College Park MD USA matysiak@umd.edu; c Department of Chemistry & Biochemistry, University of Maryland College Park MD USA; d Center for Computational Biology, Flatiron Institute New York NY USA; e United States Department of Veterans Affairs, VA Maryland Health Care System Baltimore MD USA; f Department of Microbiology and Immunology, University of Maryland School of Medicine Baltimore MD USA; g Marlene and Stewart Greenebaum Cancer Center Baltimore MD USA

## Abstract

Despite the importance of biophysical cues in tuning the immune response, the connections between these cues and immunological outcomes are poorly understood in the context of immunotherapies. To study these connections, our lab designed therapeutic complexes that are self-assembled from peptide antigens modified with cationic amino acid residues and anionic, nucleic acid-based modulatory cues. We utilized the self-assembly platform as a tool to understand how tuning the biophysical properties of immune signals impacts molecular interactions during self-assembly. Here, we implemented molecular dynamics simulations as a tool to study how molecular interactions between cationic peptides and anionic modulatory cues change as a function of peptide design. Using temperature replica exchange molecular dynamics, we compare molecular contacts – including hydrogen bonding and salt bridges – across a library of peptide sequences that are mistakenly attacked during autoimmune disease. We show that peptides with higher cationic charge and peptides anchored with arginine residues form more electrostatic interactions during self-assembly than peptides with lower cationic charge and peptides anchored with lysine residues, respectively. Surface plasmon resonance studies revealed that in addition to the type of anchored amino acid residue, the distribution of charge across the peptide also impacts the binding affinity of self-assembled immune cues. *In vitro* primary cell studies using these same antigen designs revealed signaling that was likewise sensitive to the total charge, charge distribution, and type of anchored amino acid residues within the therapeutic complexes. Taken together, these insights help intuit how to modify biophysical cues to self-assemble a range of peptide antigens for distinct disease targets. This granular understanding of nanomaterial-immune interactions contributes to more rational immunotherapy design.

## Abstract 2

Molecular dynamics simulations were implemented to study self-assembly of cationic peptide antigens and an anionic modulatory cue to inform rational immunotherapy design.

## Graphical abstract

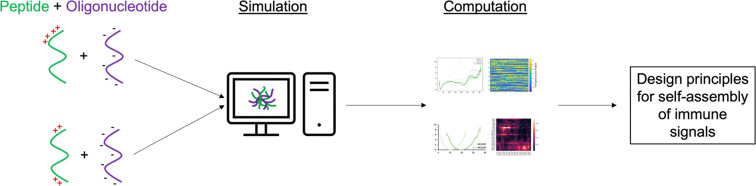

**New concepts**

We utilized self-assembly as a platform to understand how tuning the biophysical properties of immune signals impacts molecular interactions. This included molecular dynamics simulations to study how molecular interactions between cationic peptides and anionic modulatory cues change as a function of peptide design. Using temperature replica exchange molecular dynamics, we compare molecular contacts – including hydrogen bonding and salt bridges – across a library of peptide sequences mistakenly attacked during autoimmunity. We show peptides with higher cationic charge and peptides anchored with arginine residues form more electrostatic interactions during self-assembly than peptides with lower cationic charge and peptides anchored with lysine residues. Surface plasmon resonance studies revealed that in addition to the type of anchored amino acid residue, the distribution of charge across the peptide also impacts the binding affinity of self-assembled cues. *In vitro* cell studies using these same antigen designs revealed signaling that was likewise sensitive to total charge, charge distribution, and type of anchored amino acid residues within the therapeutic complexes. Taken together, these insights help intuit how to modify biophysical cues to self-assemble a range of peptide antigens for distinct disease targets. This granular understanding of nanomaterial-immune interactions contributes to more rational immunotherapy design.

## Introduction

Research at the interface of biomaterials and immunology has revealed that the biophysical properties of scaffolds, particles, and other carriers of vaccines and immunotherapies impact immunological outcomes.^1–6^ For example, the mechanisms cells use during uptake and processing of immune cues are impacted by the size, shape, surface charge, and hydrophobicity of particles carrying these signals.^1,2,7–14^ Likewise, the density of signals on or in nanoparticles can impact important immunological processes required for therapeutic efficacy.^4,5,15–18^ All of these observations indicate that biophysical properties are important design parameters to consider when formulating immunotherapies. Yet, the links between biophysical properties and immune outcomes are poorly defined. Elucidating these links could inform precisely what combination of immune cues and biophysical properties are necessary to direct immune outcomes that help fight disease.

One area where such understanding could be transformative is autoimmunity – diseases in which self-molecules identified as antigens are mistakenly recognized and attacked by the immune system. For example, during multiple sclerosis (MS), antigens found in the myelin that insulates the central nervous system are attacked, leading to loss of motor function. An experimental strategy to combat autoimmune disease involves co-delivering antigens attacked during these diseases (identified as self-antigens) with an immune cue to redirect the response to the antigens.^4,19–22^ These strategies seek to direct the processing of immune cues and the interactions between immune cells to bias responses away from inflammation and toward regulatory responses, known as tolerance, that help stop autoimmune attack. With this strategy in mind, our lab developed self-assembled complexes composed of a self-antigen attacked during MS – myelin oligodendrocyte glycoprotein (MOG), and GpG, an oligonucleotide sequence that inhibits toll-like receptor (TLR) signaling;^1,4^ TLRs are innate inflammatory cascades triggered during many autoimmune processes. MOG peptide antigens were anchored with cationic arginine or lysine residues to increase positive charge of the MOG peptide sequence and to facilitate self-assembly with negatively charged GpG *via* electrostatic interactions. This resulted in a high density nanoparticle assembly composed entirely of self-antigen and regulatory cue. We previously demonstrated that MOG and GpG self-assembled with a higher binding affinity when MOG peptides were anchored with increased cationic charge.^1^ This affinity change negatively impacted the availability of GpG to inhibit inflammatory signaling in immune cells, demonstrating the importance of biophysical properties on immune processing.

The ability to leverage such principles for immunotherapy design would be enabled by a systematic understanding of how specific parameters impact biophysical properties of immunotherapies. Thus, we sought to study how molecular interactions between MOG peptide and GpG oligonucleotide change as a function of peptide design; this information will reveal which features cause differences in binding affinity and provide predictive insight for future therapeutic designs. However, the initial self-assembly step is challenging to study experimentally due to the short timescale of assembly. From this perspective, biomolecular simulations can be a powerful alternative to explore these self-assembly processes. Conventional molecular dynamics simulations at all-atom resolution have limited applications for studying self-assembly because these simulations can become trapped in local free energy minimum conformations. Thus, traditional molecular dynamics approaches do not guarantee an exhaustive sampling in the conformational space of self-assembled biomolecules. To overcome this limitation, enhanced sampling methods such as replica exchange molecular dynamics (REMD) have been proposed.^23,24^ REMD combines molecular dynamics simulations with a Monte Carlo algorithm to expedite conformational swapping. During a REMD simulation, several replicas of the same system are simulated in parallel using molecular dynamics simulations at different temperatures. Periodically, swaps between neighboring replicas are attempted with a predefined probability of success. Over the course of the simulation, this method can overcome high free energy barriers and sufficiently explore the conformational space.

Here we employed REMD simulations to show that during self-assembly, MOG peptides with higher cationic charge and anchored with arginine residues form more total contacts, hydrogen bonds, and salt-bridges with GpG oligonucleotide than MOG peptides with lower cationic charge and anchored with lysine residues, respectively. *In vitro* cell studies indicated that a more concentrated charge distribution, achieved by anchoring MOG peptide on only the C terminus, may be more beneficial for delivery of signals to immune cells than a more dispersed distribution, achieved by anchoring MOG peptide on both the N and C termini. These insights help inform rational design of self-assembling immunotherapies for distinct disease targets.

## Results and discussion

### Self-assembly of distinct MOG peptide antigen sequences with GpG oligonucleotide were simulated by REMD

We hypothesized that the differences in binding affinity between MOG and GpG that we observed in previous studies occurred due to two factors: (1) MOG peptides anchored with arginine residues facilitate more electrostatic interactions with GpG than MOG peptides anchored with lysine residues, and (2) MOG peptides formed more molecular interactions with GpG as additional cationic amino acid residues were anchored to the peptide sequence. To test these hypotheses, we designed five MOG peptides (MOGK_2_, KMOGK, MOGR_2_, MOGK_9_, MOGR_9_) to simulate the self-assembly of MOG peptide and GpG oligonucleotide (Table 1). We simulated self-assembly of MOGK_2_, MOGR_2_, MOGK_9_, and MOGR_9_ with GpG to study how molecular interactions between MOG and GpG changed when altering total charge and type of anchored amino acid residue. We also simulated self-assembly of KMOGK with GpG and compared it to MOGK_2_ to study how charge distribution in the peptide, while total charge was held constant, influenced molecular interactions during self-assembly (Table 1).

**Table 1:** Sequence and total charge of MOG peptides and GpG oligonucleotide

Signal	Sequence	Total charge
MOGK_2_	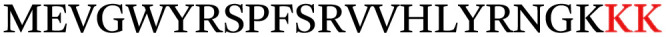	+5
KMOGK	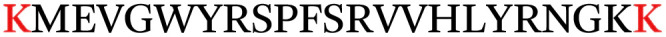	+5
MOGK_9_	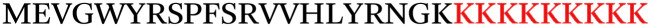	+12
MOGR_2_	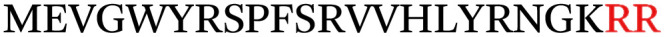	+5
MOGR_9_	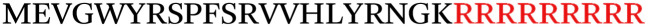	+12
GpG	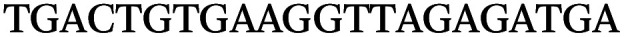	−22

After simulations were completed, we performed two quality control analyses to confirm convergence before interpreting the data. The data and methodology of the quality control steps are included in the SI (Fig. S1 and 2). Atomistic renderings of representative MOG/GpG self-assembled configurations near the potential of mean force (PMF) minimum are provided in in the SI as well (Fig. S3).

### MOG peptide and GpG oligonucleotide self-assemble into 13 Å conformations

**Fig. 1:**
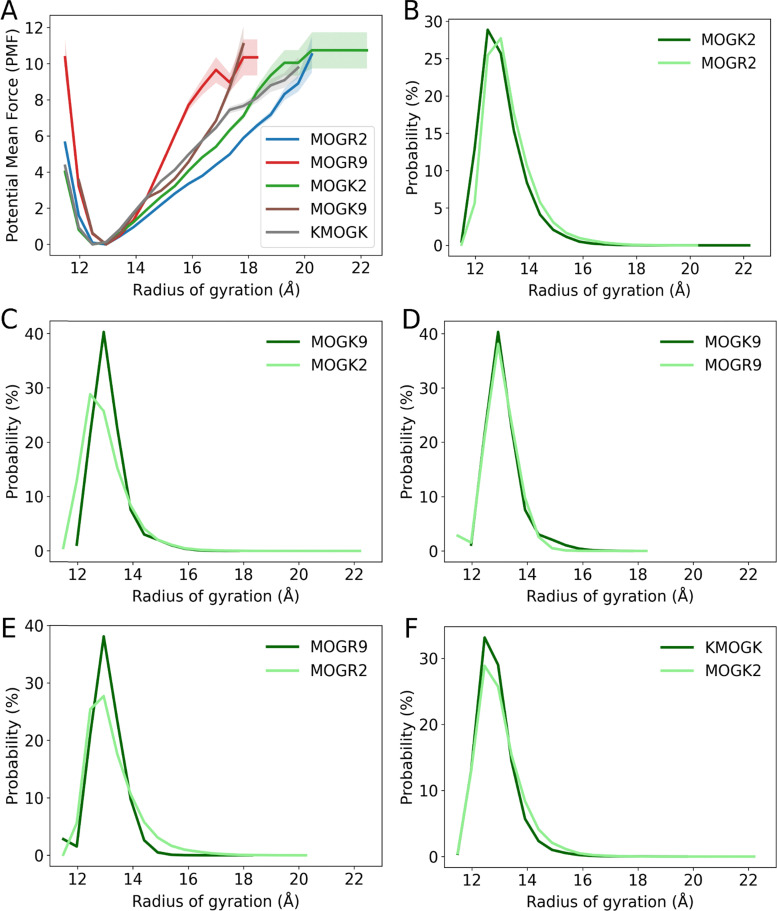
(A) Free energy profiles, measured as potential of mean force (PMF) in kT units, as a function of the radius of gyration, for complexes of different MOG peptides with GpG. (B)–(F) Comparison of the probability distributions for MOG/GpG complexes across different radii of gyration for (B) MOGK_2_*vs.* MOGR_2_, (C) MOGK_2_*vs.* MOGK_9_, (D) MOGK_9_*vs.* MOGR_9_, (E) MOGR_2_*vs.* MOGR_9_, and (F) MOGK_2_*vs.* KMOGK. Data were collected over the converged portions for each MOG peptide variant.

After confirming convergence, we examined the conformations of MOG/GpG complexes, using differences in the radius of gyration (*R*_g_) of MOG/GpG complexes to distinguish between different conformations. Comparing all five peptide simulations, the PMF profiles show that each MOG/GpG complex reaches its lowest free-energy state at an *R*_g_ near 12.5–13 Å (Fig. 1A). This indicates that neither the peptide length nor the total charge impacted the size of the most stable conformation. The additional cationic residues are flexible and electrostatically collapse onto the anionic GpG rather than extending the complex (Fig. S3). Because *R*_g_ scales sublinearly with chain length,^27,28^ the modest length differences across our peptide library are not expected to appreciably shift the size of the equilibrium complex.

We did observe differences in the probabilities of MOG/GpG complexes to be found in their lowest free energy conformations. Probabilities were calculated by dividing the number of frames found in each *R*_g_ bin by the total number of frames being analyzed. For this analysis, different converged time intervals were analyzed for each MOG peptide simulation such that the total number of frames being analyzed were all within 1% of each other. MOGK_2_ exhibits a very similar *R*_g_ distribution to MOGR_2_, with both distributions dominated by a compact peak near 13 Å (Fig. 1B). In contrast, MOGK_9_ shows a higher and narrower peak near 13 Å than MOGK_2_, indicating a higher preference for the compact configuration (Fig. 1C). Comparing MOGK_9_ and MOGR_9_, both distributions remain centered near 13 Å, with only modest differences in peak height and tail behavior (Fig. 1D). MOGR_9_ shows a more sharply peaked compact-state distribution than MOGR_2_, whereas MOGR_2_ samples a broader range of *R*_g_ values with a comparatively larger high-*R*_g_ tail (Fig. 1E). Finally, KMOGK and MOGK_2_ are both dominated by the compact state near 13 Å (Fig. 1F).

Overall, *R*_g_ is useful for classifying the ensemble by compactness and identifying the dominant compact state across all variants, but it is not sufficient on its own to explain the binding affinity between MOG peptides and GpG. For example, experimental data indicates that MOG peptides with more cationic charge exhibit a higher binding affinity for GpG than MOG peptides with less cationic charge.^1^ However, all MOG/GpG complexes were found in near 13 Å conformations, regardless of total MOG peptide charge. Thus, to have predictive power with molecular dynamics, additional insights are necessary that explain the differences in experimentally measured binding affinity between differentially charged peptide molecules.

### Peptide charge, anchored residue, and charge density alter interaction profiles between MOG and GpG

**Fig. 2:**
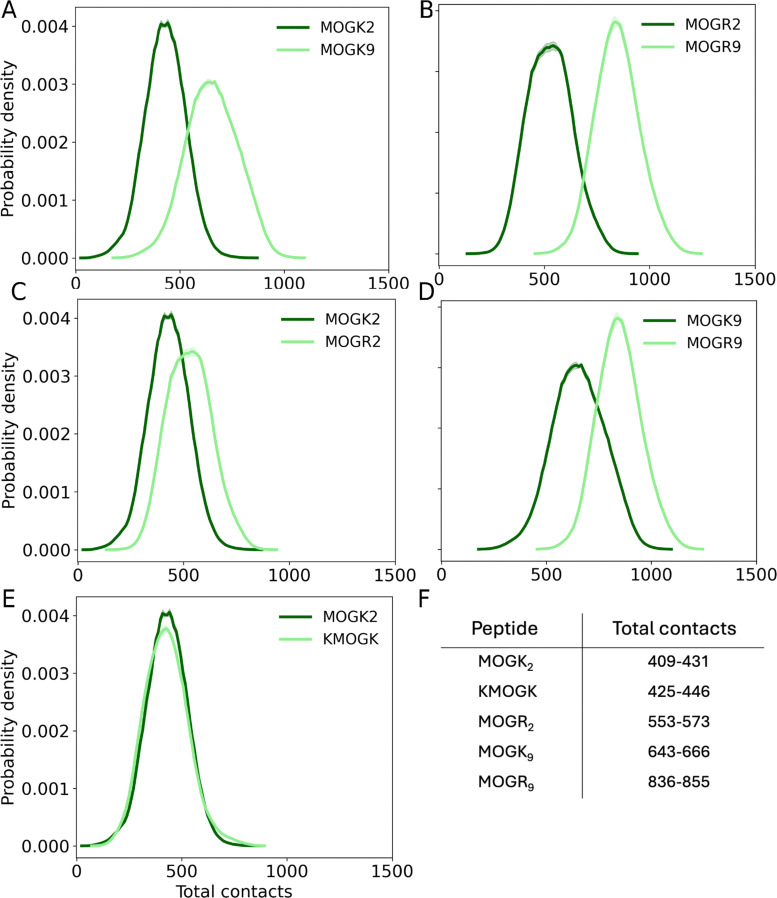
(A)–(E) Probability density distributions of total contacts formed between MOG peptide variants and GpG: (A) MOGK_2_*vs.* MOGK_9_, (B) MOGR_2_*vs.* MOGR_9_, (C) MOGK_2_*vs.* MOGR_2_, (D) MOGK_9_*vs.* MOGR_9_, and (E) MOGK_2_*vs.* KMOGK. (F) Total contacts formed between each MOG peptide and GpG.

To better understand how the conformational differences correspond to binding affinity between MOG peptides and GpG, we next analyzed how many contacts are formed during self-assembly as a function of peptide design. When comparing MOGK_2_ to MOGK_9_ (Fig. 2A) and MOGR_2_ to MOGR_9_ (Fig. 2B), we observed large differences in the total contacts formed within MOG/GpG complexes. Compared to MOGK_9_ and MOGR_9_, MOGK_2_ (Fig. 2A and F) and MOGR_2_ (Fig. 2B and F), respectively, formed fewer contacts with GpG. This can be explained through electrostatics, with more cationic charges on MOGK_9_ and MOGR_9_ compared to MOGK_2_ and MOGR_2_. Interestingly, when comparing MOGK_2_ to MOGR_2_ (Fig. 2C and F) and MOGK_9_ to MOGR_9_ (Fig. 2D and F), we observed that MOG peptides anchored with arginine residues formed more contacts than MOG peptides anchored with lysine residues. These data align well with the experimental results that indicate arginine confers a higher binding affinity than lysine during electrostatic self-assembly.^1^

To characterize the specific contacts formed between amino acids of the MOG peptide sequence and nucleotides of the GpG oligonucleotide sequence, we used contact maps. Towards this, we computed all contacts formed between amino acids of MOG and nucleotides of GpG and then calculated what frequency each amino acid and nucleotide pair was of the total contacts formed. Using this data, heatmaps were then generated to visualize frequencies of each amino acid and nucleotide contact relative to all contacts formed.

**Fig. 3:**
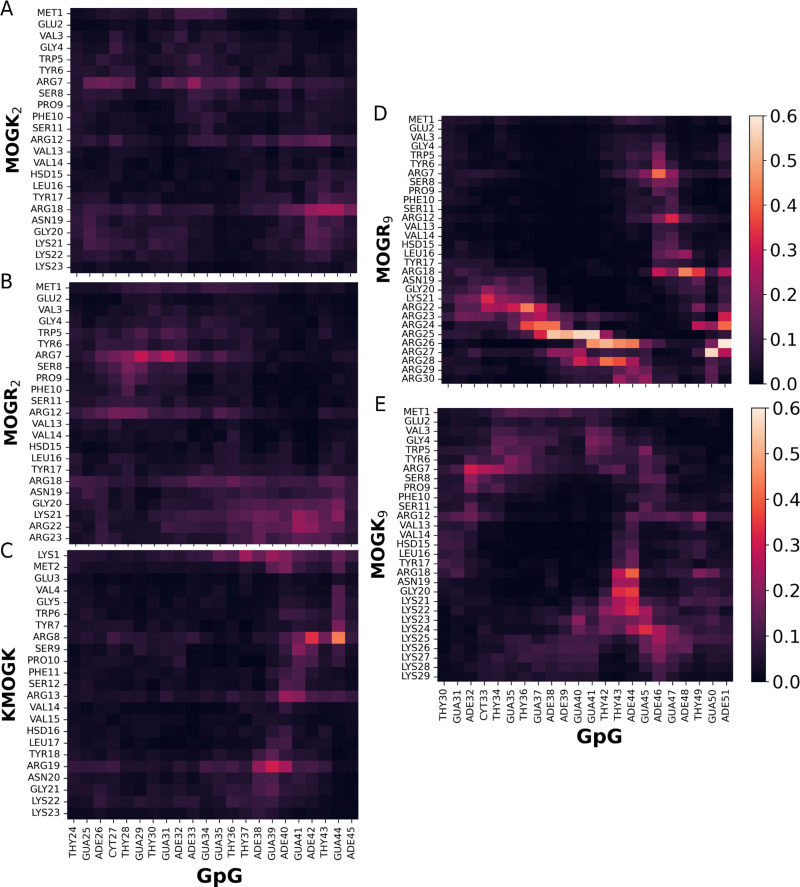
Contact frequency heatmaps between individual amino acid residues of MOG peptide variants (*y*-axis) and nucleotides of GpG (*x*-axis) for (A) MOGK_2_, (B) MOGR_2_, (C) KMOGK, (D) MOGR_9_, and (E) MOGK_9_. Color intensity indicates contact frequency, with warmer colors (white/orange) representing higher frequency and cooler colors (purple/black) representing lower frequency.

Fig. 3 illustrates differences in the types of contacts that drive self-assembly between MOG peptides and GpG. MOGK_2_ (Fig. 3A) forms high-frequency contacts primarily through basic residues (ARG7, ARG12, and ARG18), which show contact frequencies of 0.15–0.25 distributed across multiple GpG nucleotides. MOGR_2_ exhibits strong contacts near the 3' end of GpG, with contact frequency of 0.20–0.25 formed by C-terminal basic residues (Fig. 3B). Additional interactions from N-terminal basic residues (ARG7) create a second hotspot in the 5′ half and weaker contacts across the central nucleotides. Intriguingly, anchoring the MOG peptide with arginine residues on the C terminus facilitated more contacts with the 3′ end of GpG. When the MOG peptide was anchored with lysine residues on both the N and C terminus (Fig. 3C), contacts with a relative frequency of 0.2 or higher were not seen at the 5′ end of GpG like they were with MOGK_2_. These data suggest that molecular interactions between MOG peptide and GpG are influenced by both the type of anchored amino acid residue and the distribution of charge on the peptide sequence.

Analyzing the contact heatmaps of MOGR_9_ (Fig. 3D) and MOGK_9_ (Fig. 3E), the differences in interaction profiles are even more stark compared to the lower charge peptides. The highest frequency contacts of MOGR_9_ and MOGK_9_ range from ∼0.5–0.6, which is approximately double the ∼0.2–0.3 range of MOGK_2_ and MOGR_2_. The heatmaps of MOGK_9_ and MOGR_9_ contain broad areas with contact frequencies less than 0.1 and tight zones of high frequency contacts (∼0.4–0.6). The higher total charge and charge density of the arginine and lysine tails of MOGK_9_ and MOGR_9_ facilitate fewer but high-frequency contacts with GpG. This contrasts with the heatmaps of MOGK_2_, MOGR_2_, and KMOGK, which have broad areas where contact frequencies are ∼0.1–0.3. Thus, compared to the higher charge peptides, the lower charge peptides facilitated more types of contacts between MOG peptide and GpG, but these contacts occurred at lower overall frequencies.

Considering these results with the surface plasmon resonance data described previously,^1^ we see an intriguing association between experimentally measured binding affinity and computationally modeled molecular interaction. Self-assembly of MOG peptides characterized with a lower binding affinity for GpG (MOGK_2_ and MOGR_2_) were driven by non-specific interactions that were weaker and occurred at lower frequencies. Self-assembly of MOG peptides characterized with a higher binding affinity for GpG (MOGK_9_ and MOGR_9_) were driven by fewer types of contacts that were stronger and occurred at higher frequencies. This kind of insight highlights the potential of using computational modeling to help design self-assembly of immune signal with defined biophysical characteristics, such as high or low binding affinity. For example, one may need to design immune signals with a binding affinity that's high enough to achieve self-assembly but low enough to remain bioactive and effectively exert their biological function. Another context may require more tightly bound immune signals to facilitate better protection from environmental factors such as enzymatic degradation. To achieve such granularity and predictive power, it will be important to understand not only where and how many contacts are forming, but also the specific type of molecular interactions that influence differences in biophysical characteristics. One commonality with all MOG peptides was that the highest frequency contacts always included arginine or lysine residues. This points to the importance of charged amino acids in driving molecular interactions during electrostatic self-assembly of MOG peptides and GpG oligonucleotide.

### Arginine residues facilitate more electrostatic interactions than lysine residues

**Fig. 4:**
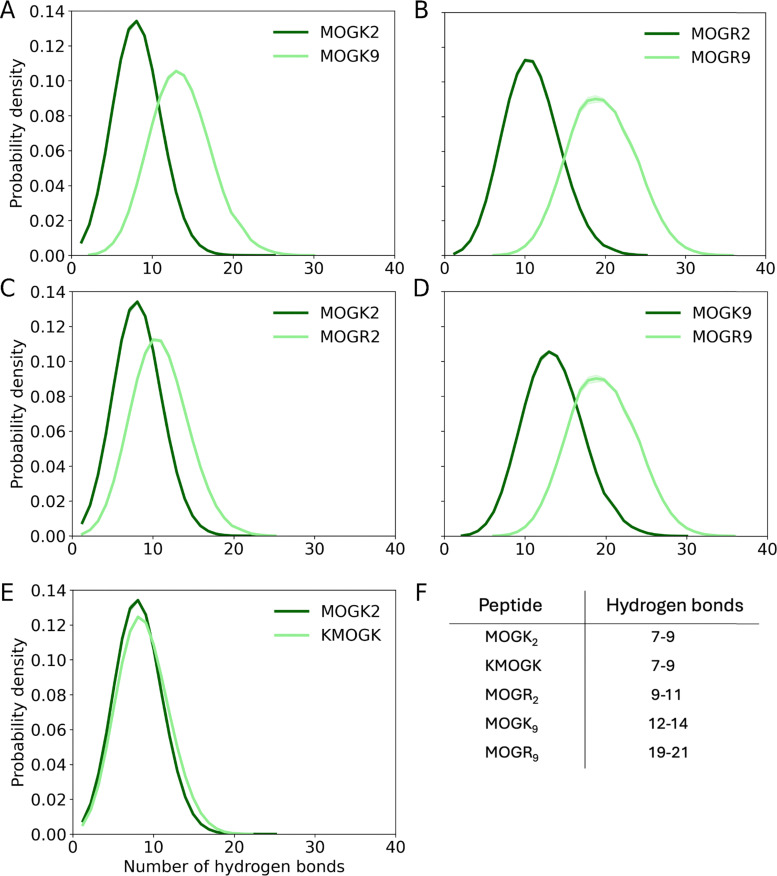
(A)–(E) Probability density distributions of hydrogen bonds formed between MOG peptide variants and GpG: (A) MOGK_2_*vs.* MOGK_9_, (B) MOGR_2_*vs.* MOGR_9_, (C) MOGK_2_*vs.* MOGR_2_, (D) MOGK_9_*vs.* MOGR_9_, and (E) MOGK_2_*vs.* KMOGK. (F) Number of hydrogen bonds formed between each MOG peptide variant and GpG at their respective lowest free energy conformations.

Since self-assembly of MOG peptides and GpG oligonucleotide is driven by electrostatics, we analyzed how molecular interactions driven by charge polarity changed as a function of MOG peptide design. We started by computing how many hydrogen bonds were formed between MOG and GpG in the different simulations and analyzed how the number of hydrogen bonds changed with different MOG peptide designs. When comparing MOGK_2_*vs.* MOGK_9_ (Fig. 4A and F) and MOGR_2_*vs.* MOGR_9_ (Fig. 4B, 4F), we observed MOGK_2_ and MOGR_2_ formed 5 and 10 fewer hydrogen bonds, respectively, with GpG in their lowest free energy states. These results indicate that MOG peptides with higher cationic charge and longer sequence lengths formed more hydrogen bonds compared to MOG peptides with lower cationic charge and shorter sequence lengths. Moreover, when comparing MOGK_2_*vs.* MOGR_2_ (Fig. 4C and F) and MOGK_9_*vs.* MOGR_9_ (Fig. 4D and F), we observed that peptides with trailing lysine formed fewer hydrogen bonds with GpG in their lowest free energy states. These results indicate that MOG peptides anchored with arginine residues formed more hydrogen bonds with GpG than MOG peptides anchored with lysine residues. This observation can be explained by the presence of two additional hydrogen bonding groups on the arginine sidechain (–NH_2_, =NH) that are not present on the lysine sidechain. The extra hydrogen bonding groups facilitate more interactions between MOG and GpG despite the equivalent overall charge and sequence length of the two MOG peptides. When comparing MOGK_2_*vs.* KMOGK (Fig. 4E and F), we observed MOGK_2_ formed a similar number of hydrogen bonds with GpG as KMOGK in its lowest free energy states. This data indicates the number of hydrogen bonds was not impacted when distributing the charge by anchoring lysine on both the N and C terminus (KMOGK), as opposed to just the C terminus (MOGK_2_). However, the right tail of KMOGK is slightly broader than MOGK_2_ (Fig. 4E), suggesting that distributing the charge between N and C termini results in a higher probability of maximizing hydrogen bonding during self-assembly.

**Fig. 5:**
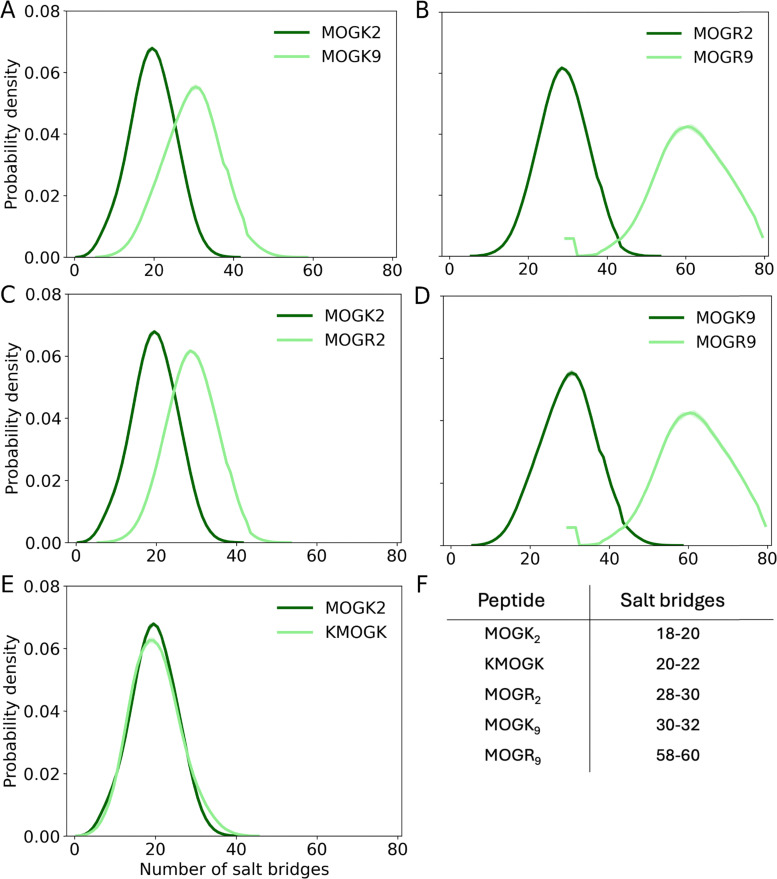
(A)–(E) Probability density distributions of salt bridges formed between MOG peptide variants and GpG: (A) MOGK_2_*vs.* MOGK_9_, (B) MOGR_2_*vs.* MOGR_9_, (C) MOGK_2_*vs.* MOGR_2_, (D) MOGK_9_*vs.* MOGR_9_, and (E) MOGK_2_*vs.* KMOGK. (F) Number of salt bridges formed between each MOG peptide variant and GpG at their respective lowest free energy conformations.

Because MOG and GpG are designed to self-assemble *via* electrostatic interactions, we also analyzed formation of salt bridges and how the number of salt bridges changed with different MOG peptide designs. Salt bridges were defined as a contact between a nitrogen on arginine, lysine, or histidine on the MOG peptide and an oxygen on the GpG oligonucleotide. In their lowest free energy states, MOGK_2_ and MOGR_2_ formed fewer salt bridges than MOGK_9_ (Fig. 5A and F) and MOGR_9_ (Fig. 5B and F), respectively. These results indicate that MOG peptides with higher cationic charge and longer sequence lengths formed more salt bridges compared to MOG peptides with lower cationic charge and shorter sequence lengths. Similarly, when comparing MOGK_2_*vs.* MOGR_2_ (Fig. 5C and F) and MOGK_9_*vs.* MOGR_9_ (Fig. 5D and F), we observed MOGK_2_ and MOGK_9_ formed 10 and 28 fewer salt bridges, respectively, with GpG in their lowest free energy states. When comparing MOGK_2_*vs.* KMOGK (Fig. 5E and F), we observed MOGK_2_ and KMOGK formed similar number of salt bridges with GpG in their lowest free energy states. These results indicate that MOG peptides anchored with arginine residues formed more salt bridges with GpG than MOG peptides anchored with lysine residues. This result can be explained by the presence of two additional nitrogen atoms on the arginine sidechain that are not present on the lysine sidechain. The number of salt bridges was not impacted when distributing the charge by anchoring lysine on both the N and C terminus (KMOGK), as opposed to just the C terminus (MOGK_2_). Similarly to hydrogen bonds, the right tail of KMOGK is slightly broader than MOGK_2_ (Fig. 5E). These data suggest that distributing the charge between N and C termini results in a higher probability of maximizing salt bridge interactions during self-assembly.

Our analysis of electrostatic interactions reveals that arginine residues facilitate a greater number of hydrogen bonds and salt bridges, compared to lysine residues, during self-assembly of MOG peptide and GpG oligonucleotide. Given that self-assembly of MOG and GpG is driven by electrostatics, the ability of arginine to form more hydrogen bonds and salt bridges than lysine likely conferred a higher binding affinity to MOG peptides anchored with arginine residues during the surface plasmon resonance studies described previously.^1^ Similarly, MOG peptides with higher total charge (MOGK_9_ and MOGR_9_) facilitated a greater number of hydrogen bonds and salt bridges compared to MOG peptides with lower total charge (MOGK_2_ and MOGR_2_). However, with this analysis, it is difficult to distinguish between the effect of the inherent peptide charge and the presence of more amino acids to facilitate more electrostatic interactions. A different modeling approach, like coarse-grained methods, may be more suitable to elucidate the effects of overall charge balance on self-assembly. The advantage of coarse-grained simulations is the ability to model the self-assembly of multiple peptides and oligonucleotides in a single system. By altering the number of peptides and oligonucleotides in the system, we could study the effect that charge balance in solution has on self-assembly of MOG and GpG into complexes.

Anchoring MOG with lysine residues on both the N and C terminus did not result in differences in total number of hydrogen bonds or salt bridges compared to anchoring MOG with lysine residues on only the C terminus. However, the probability of finding the MOG/GpG complex in a conformation with hydrogen bonds and salt bridges was slightly higher when the positive charge was distributed between both N and C termini (KMOGK) compared to when the positive charge was clustered on the C terminus (MOGK_2_). A more systematic analysis of charge distribution by including peptides with different variations of K/R at both ends of MOG can be more instructive towards correlating charge distribution and binding affinity.

### Effects of distributing charge density are different when comparing lysine to arginine

Beyond specific electrostatic interactions, a contact map can be a useful indicator of binding affinity as it curates both specific and non-specific interactions. When comparing MOGK_2_ and MOGR_2_ (Fig. 3A and B), we observed that MOGR_2_ contains fewer low-frequency and clearer high-frequency regions than MOGK_2_. Given that MOGR_2_ binds GpG more strongly than MOGK_2_,^1^ greater variations in the types of contacts between MOG and GpG could be another indicator of relatively higher binding affinity. When comparing MOGK_2_ and KMOGK (Fig. 3A and C), we observed that KMOGK exhibits stronger localized hotspots, particularly closer to the 3′ end, despite having the same net charge and sequence length as MOGK_2_. Together, this can suggest that the identity and placement of charged residues can reshape the contact footprint and may contribute to differences in binding affinity for GpG.

**Fig. 6:**
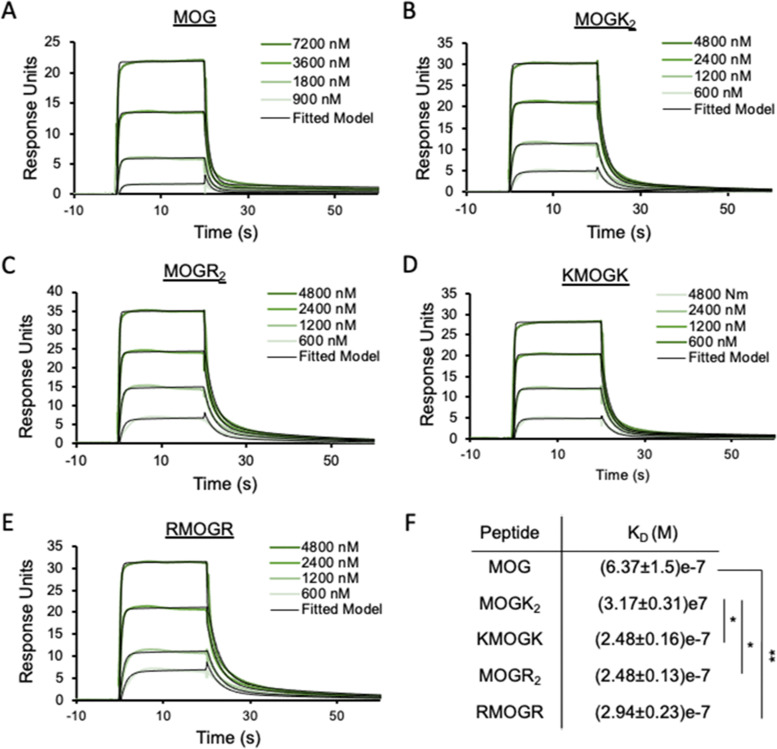
Representative kinetic curves from one surface plasmon resonance experiment are shown for (A) MOG, (B) MOGK_2_, (C) MOGR_2_, (D) KMOGK, and (E) RMOGR. (F) Average *K*_D_ values (*n* = 3) calculated for binding between MOG peptides and GpG. Statistics were analyzed by One way ANOVA with Tukey post-test to correct for multiple comparisons. *p < 0.05, **p < 0.01.

To test this initial hypothesis, we used surface plasmon resonance to measure the binding affinity between GpG and MOG (Fig. 6A), MOGK_2_ (Fig. 6B), MOGR_2_ (Fig. 6C), KMOGK (Fig. 6D), and RMOGR (Fig. 6E). MOG peptides were flowed over a sensor chip coated with GpG to measure the association and dissociation rates between peptides and GpG. The kinetic binding curves were then fitted to a two-state binding model and the dissociation rate constants (*K*_D_) between MOG peptides and GpG were calculated. A two-state binding model was chosen with the expectation that the peptide-oligonucleotide complex is formed after an initial binding event, followed by a subsequent solvent exclusion-like step to readjust to an entropically favorable conformation.^25^

When anchoring MOG with either arginine or lysine, *K*_D_ values decreased significantly compared to the native MOG peptide (Fig. 6F). This observation indicated that the binding affinity between MOG and GpG increased when MOG exhibited a more positive charge. Based on the simulation results, native MOG peptide likely did not form as many electrostatic interactions with GpG as MOG peptide anchored with arginine or lysine. When comparing MOGK_2_ to MOGR_2_, *K*_D_ values were significantly lower when MOG was anchored with arginine residues rather than lysine residues. This is consistent with previous reports of the nature of these two residues.^1^ Since both MOGK_2_ and MOGR_2_ have the same overall charge, this finding suggests that additional factors impacted the molecular interactions between MOG peptides and GpG. Our simulation data revealed that arginine residues facilitate more hydrogen bonding and salt bridges between MOG and GpG than lysine residues, which predicts the experimental result.

Intriguingly, KMOGK exhibited a significantly lower *K*_D_ value than MOGK_2_ (Fig. 6F). These data indicate that compared to MOGK_2_, increasing charge distribution by anchoring MOG peptide with lysine on both the N and C terminus increased binding affinity with GpG. This result is in line with our initial hypothesis. The contact heatmaps indicated that KMOGK exhibits stronger localized hotspots, despite having the same net charge and sequence length as MOGK_2_ (Fig. 3A and C). The simulation data also indicated that KMOGK had a higher probability of maximizing hydrogen bonding and salt bridge interactions than MOGK_2_ (Fig. 4E and 5E). Taken together, these data indicate that when MOG is anchored with lysine residues, distributing the charge on both N and C termini increased binding affinity between MOG and GpG by increasing the probability of high frequency electrostatic interactions. This trend may be different when distributing charge with lysine residues and arginine residues. When measured by surface plasmon resonance, the binding affinity of RMOGR decreased compared to MOGR_2_ (Fig. 6F). It may be that because arginine forms more electrostatic interactions with GpG than lysine, concentrating arginine residues together could confer a higher binding affinity compared to distributing the arginine residues. This hypothesis could be investigated using REMD simulations of RMOGR and GpG.

### MOG/GpG complexes reduce inflammatory gene expression and signaling in dendritic cells

**Fig. 7:**
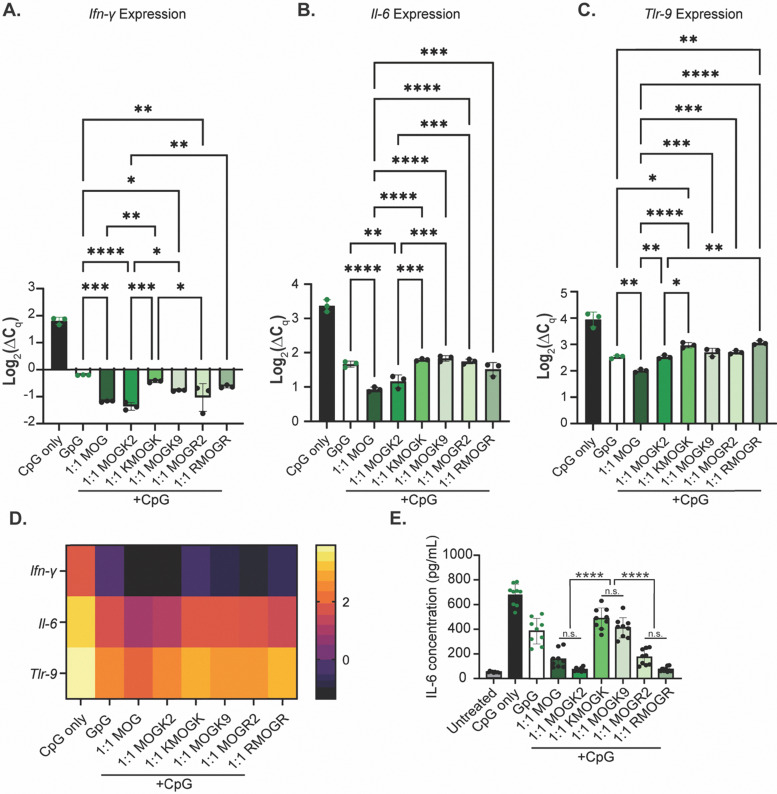
Gene expression of A) *Tlr9*, B) *IFN-γ*, and C) *Il6*, and D) ELISA data of IL-6 for DCs treated with complexes of GpG and MOG anchored with different amino acid residues. Statistics were analyzed by One way ANOVA with Tukey post-test to correct for multiple comparisons. *p < 0.05, **p < 0.01.

Finally, we tested if the trends in molecular interactions identified in the simulations correlate to specific outcomes in gene expression and protein signaling studies performed on primary immune cells *in vitro*. For the *in vitro* studies, we utilized dendritic cells (DCs) because these immune cells express high levels of toll-like receptor 9 (TLR9) that GpG can antagonize and regulate inflammatory signaling upon binding. We sought to test how anchoring MOG with cationic amino acids impacted the availability of GpG to regulate inflammatory gene expression and protein secretion when delivered to the cells as MOG/GpG complexes. The gene expression profiles of DCs treated with complexes containing GpG and different MOG peptides (MOG, MOGK_2_, KMOGK, MOGK_9_, MOGR_2_, and MOGR_9_) showed an apparent decrease in inflammatory transcripts (Fig. 7A–C). Compared to the CpG positive control, which is a TLR9 receptor agonist, the different complexes suppressed transcription of the inflammatory genes *Ifn-γ* and *Il-6*.

Interestingly, delivery of GpG in MOG/GpG complexes containing MOG, MOGK_2_, MOGK_9_, and MOGR_2_, lead to a significant decrease in gene expression of *Ifn-γ* compared to GpG alone (Fig. 7A). This result indicates a stronger suppression of inflammatory signaling by DCs when GpG was delivered in complex form compared to soluble form. This result also indicates that a concentrated charge in MOGK_2_ is more beneficial for delivering GpG to DCs than a more distributed charge in KMOGK. In this case, the lower binding affinity between MOGK_2_ and GpG may have allowed GpG to better exert its biological function upon entering the cells. This correlation is also supported by the *Il-6* expression (Fig. 7B), where only MOG/GpG and MOGK_2_/GpG complexes lead to a significant decrease compared to GpG alone, and these two peptides had the lowest binding affinity compared to the rest of the MOG peptides (Fig. 6F). As GpG is an inhibitor of the TLR-9 signaling pathway, we also measured the gene expression of *Tlr-9* in DCs in response to treatment with MOG/GpG complexes. Not surprisingly, all complexes led to a decrease in gene expression of *Tlr-9* when compared to the CpG positive control (Fig. 7C). Interestingly, only the MOG/GpG complex, which has the lowest binding affinity, had a significant decrease in *Tlr-9* compared to GpG only. Some complexes (KMOGK and RMOGR) had *Tlr-9* gene expression greater than that observed in the soluble GpG group. This could indicate that charge distribution across the MOG peptide might lead to modest activation of the TLR9 pathway compared to GpG alone.

Overall, the gene expression data indicates that the type of charge, as well as charge density on the peptide, modulates inflammatory signals in DCs (Fig. 7D), with KMOGK/GpG and RMOGR/GpG complexes having modest yet significant increases in *Ifn-γ* and *Tlr-9* gene expression profiles when compared to the GpG only control. To examine how these changes in gene expression translated to protein signaling, we used an enzyme-linked immunosorbent assay (ELISA) to quantify the release of IL-6 by DCs (Fig. 7E). IL-6 is a cytokine implicated in the onset of experimental autoimmune encephalomyelitis (EAE),^26^ a mouse model of MS. Thus, a therapy that reduces or prevents its secretion could prevent or lessen the extent of disease. We found that all tested complexes blunted IL-6 production compared to CpG positive control. This indicates that delivery of GpG in complex form can blunt secretion of inflammatory signals in an inflammatory environment. Additionally, all complex formulations caused either similar or decreased IL-6 production compared to GpG + CpG alone. This result indicates that the presence of MOG did not cause additional inflammatory signaling in cells, which is important in a therapy delivering immune signals to regulate, rather than stimulate, an immune response. In examining how the placement of cationic residues impacted IL-6 secretion, we found that where arginines were anchored (*e.g.*, MOGR_2_*vs.* RMOGR) had no significant effect compared to each other. However, changing the placement of lysine residues from MOGK_2_ to KMOGK resulted in nearly a seven-fold increase in IL-6, even though the total charge and composition of peptides was the same. This has implications for the rational design of self-assembling immune cues, as appending a small amount of lysine (or other charge group) on either side of the peptide may result in drastic differences in the secretion of key signaling proteins. Interestingly, MOGK_2_ and RMOGR were the only two peptides able to return IL-6 levels to that of untreated cells. This indicates that not only the placement but also the type of amino acid must be carefully considered when rationally designing self-assembling immune signals for immunotherapies.

## Conclusions

Molecular dynamics simulations are a powerful tool to inform design of self-assembling immunotherapies with specific biophysical properties, such as higher or lower binding affinity. The ability to understand and control these design levers will enable innovative methods of programming immune responses to combat diseases such as cancer and autoimmunity. Our studies revealed a stark contrast in molecular interactions when anchoring MOG peptides with different total number (2 *vs.* 9) and type (lysine *vs.* arginine) of cationic residues. Specifically, we revealed that MOG peptides with higher total charge or anchored with arginine residues formed more electrostatic interactions with GpG than MOG peptides with lower total charge or anchored with lysine residues, respectively. These data explain experimental measurements that indicate MOG peptides with higher total charge or anchored with arginine residues have a higher binding affinity with GpG than MOG peptides with lower total charge or anchored with lysine residues, respectively. Additional simulations with completely different peptide antigens would be useful in determining how the insights discussed here translate to different self-assembly systems. If the trends observed in our studies translate to other peptide/oligonucleotide self-assembly systems, we can leverage molecular dynamics as a screening tool for designing modular, self-assembling immunotherapies with specifically designed biophysical characteristics. Since REMD is resource intensive, other molecular simulation methods with advanced sampling protocols or reduced resolution approaches could be applied. With continued collaboration between immunoengineers and computational researchers, new and more efficient methodologies can be developed to help build the next generation of immunotherapies.

## Conflicts of interest

CMJ and RSO are employees of the VA Maryland Health Care System. The views reported in this paper do not reflect the views of the Department of Veterans Affairs or the United States Government. CMJ has equity positions with Cartesian Therapeutics, Nodal Therapeutics, Patch Bio, Aletira Therapeutics, Allometra Materials, CREATE Medicines, Antidote Tx, and Barinthus Biotherapeutics. Remaining authors declare no competing interests.

## Supplementary Material

NH-011-D6NH00215C-s001

## Data Availability

All data are available in main text or supplementary information (SI). Supplementary information: quality control analysis of TREMD simulations, atomistic representations of MOG/GpG complexes, and materials and methods section. See DOI: https://doi.org/10.1039/d6nh00215c.

## References

[cit1] Froimchuk E., Oakes R. S., Kapnick S. M., Yanes A. A., Jewell C. M. (2021). Biophysical Properties of Self-Assembled Immune Signals Impact Signal Processing and the Nature of Regulatory Immune Function. Nano Lett..

[cit2] Tsai S. J., Amerman A., Jewell C. M. (2024). Altering Antigen Charge to Control Self-Assembly and Processing of Immune Signals During Cancer Vaccination. Front. Immunol..

[cit3] Hess K. L. (2017). *et al.*, Engineering Immunological Tolerance Using Quantum Dots to Tune the Density of Self-Antigen Display. Adv. Funct. Mater..

[cit4] Hess K. L., Andorko J. I., Tostanoski L. H., Jewell C. M. (2017). Polyplexes assembled from self-peptides and regulatory nucleic acids blunt toll-like receptor signaling to combat autoimmunity. Biomaterials.

[cit5] Tsai S. J., Andorko J. I., Zeng X., Gammon J. M., Jewell C. M. (2018). Polyplex interaction strength as a driver of potency during cancer immunotherapy. Nano Res..

[cit6] Bookstaver M. L., Tsai S. J., Bromberg J. S., Jewell C. M. (2018). Improving Vaccine and Immunotherapy Design Using Biomaterials. Trends Immunol..

[cit7] Wibroe P. P. (2017). *et al.*, Bypassing adverse injection reactions to nanoparticles through shape modification and attachment to erythrocytes. Nat. Nanotechnol..

[cit8] Chen X. (2016). *et al.*, Shape-Dependent Activation of Cytokine Secretion by Polymer Capsules in Human Monocyte-Derived Macrophages. Biomacromolecules.

[cit9] Meyer R. A. (2015). *et al.*, Biodegradable nanoellipsoidal artificial antigen presenting cells for antigen specific T-cell activation. Small.

[cit10] Sunshine J. C., Perica K., Schneck J. P., Green J. J. (2014). Particle shape dependence of CD8+ T cell activation by artificial antigen presenting cells. Biomaterials.

[cit11] Hardy C. L. (2013). *et al.*, Differential uptake of nanoparticles and microparticles by pulmonary APC subsets induces discrete immunological imprints. J. Immunol..

[cit12] Kumar S., Anselmo A. C., Banerjee A., Zakrewsky M., Mitragotri S. (2015). Shape and size-dependent immune response to antigen-carrying nanoparticles. J. Controlled Release.

[cit13] Moyano D. F. (2012). *et al.*, Nanoparticle hydrophobicity dictates immune response. J. Am. Chem. Soc..

[cit14] Miura R., Sawada S. I., Mukai S. A., Sasaki Y., Akiyoshi K. (2020). Antigen Delivery to Antigen-Presenting Cells for Adaptive Immune Response by Self-Assembled Anionic Polysaccharide Nanogel Vaccines. Biomacromolecules.

[cit15] Pompano R. R. (2014). *et al.*, Titrating T-cell Epitopes Within Self-Assembled Vaccines Optimizes CD4+ Helper T Cell and Antibody Outputs. Adv. Healthcare Mater..

[cit16] Singha S. (2017). *et al.*, Peptide-MHC-based nanomedicines for autoimmunity function as T-cell receptor microclustering devices. Nat. Nanotechnol..

[cit17] Bookstaver M. L., Hess K. L., Jewell C. M. (2018). Self-Assembly of Immune Signals Improves Codelivery to Antigen Presenting Cells and Accelerates Signal Internalization, Processing Kinetics, and Immune Activation. Small.

[cit18] Bridgeman C. J., Shah S. A., Oakes R. S., Jewell C. M. (2023). Dissecting regulatory T cell expansion using polymer microparticles presenting defined ratios of self-antigen and regulatory cues. Front. Bioeng. Biotechnol..

[cit19] Froimchuk E., Carey S. T., Edwards C., Jewell C. M. (2020). Self-Assembly as a Molecular Strategy to Improve Immunotherapy. Acc. Chem. Res..

[cit20] Oakes R. S., Froimchuk E., Jewell C. M. (2019). Engineering Biomaterials to Direct Innate Immunity. Adv. Ther..

[cit21] Tostanoski L. H. (2016). *et al.*, Design of Polyelectrolyte Multilayers to Promote Immunological Tolerance. ACS Nano.

[cit22] Oakes R. S. (2021). *et al.*, Exploiting Rational Assembly to Map Distinct Roles of Regulatory Cues during Autoimmune Therapy. ACS Nano.

[cit23] Qi R., Wei G., Ma B., Nussinov R. (2018). Replica Exchange Molecular Dynamics: A Practical Application Protocol with Solutions to Common Problems and a Peptide Aggregation and Self-Assembly Example. Methods Mol. Biol..

[cit24] R Z. (2007). Replica exchange molecular dynamics method for protein folding simulation. Methods Mol. Biol..

[cit25] Ferreiro D. U., de Prat-Gay G. (2003). A protein-DNA binding mechanism proceeds through multi-state or two-state parallel pathways. J. Mol. Biol..

[cit26] Sanchis P. (2020). *et al.*, Interleukin-6 Derived from the Central Nervous System May Influence the Pathogenesis of Experimental Autoimmune Encephalomyelitis in a Cell-Dependent Manner. Cells.

[cit27] Watson M. C., Curtis J. E. (2014). Probing the Average Local Structure of Biomolecules Using Small-Angle Scattering and Scaling Laws. Biophys. J..

[cit28] Nygaard M. (2017). *et al.*, An Efficient Method for Estimating the Hydrodynamic Radius of Disordered Protein Conformations. Biophys. J..

