# Supplementary material for: Integrating molecular dynamics simulations to enable rational assembly of immune signals for immunotherapy
Source: Nanoscale Horiz. 2026 May 30;11(8):2191–203. doi: 10.1039/d6nh00215c (PMC13267778; doi:10.1039/d6nh00215c)
Supplement: NH-011-D6NH00215C-s001 [file NH-011-D6NH00215C-s001.pdf]

Supplementary Information (SI) for Nanoscale Horizons.
This journal is © The Royal Society of Chemistry 2026

Integrating Molecular Dynamics Simulations to Enable Rational Assembly of Immune Signals for Immunotherapy

Supplementary Information

#Eugene Froimchuk[1,2], #Meenal Jain[3], Abhilash Sahoo[4], Camilla Edwards[1,2], Shrey Shah[1,2], Robert S. Oakes[1,2,5], *Silvina Matysiak[2], and *Christopher M. Jewell[1,5-7]

# These authors contributed equally

* Correspondence to: cmjewell@umd.edu
matysiak@umd.edu

Affiliations

1 Robert E. Fischell Institute for Biomedical Devices; College Park, MD, USA.
2 Fischell Department of Bioengineering, University of Maryland, College Park, MD, USA.
3 Department of Chemistry & Biochemistry, University of Maryland, College Park, MD, USA.
4 Center for Computational Biology, Flatiron Institute; New York, NY, USA.
5 United States Department of Veterans Affairs, VA Maryland Health Care System; Baltimore, MD, USA.
6 Department of Microbiology and Immunology, University of Maryland School of Medicine; Baltimore, MD, USA.
7 Marlene and Stewart Greenebaum Cancer Center; Baltimore, MD, USA.

Quality Control Analyses of TREMD Simulations

First, we computed the potential of mean force (PMF) as a function of the radius of gyration for the MOG/GpG complex over successive cumulative time windows (0–100, 0–200, 0–300, and 0–400 ns) for each peptide variant, with the final window adjusted to the full trajectory length for $MOGR_2$ (0–350 ns) and $MOGR_9$ (0–300 ns). Convergence was assessed by comparing PMF profiles across these time windows. Overlapping PMF profiles indicate simulation convergence. Determining where the simulation converges is important for identifying the time interval over which computational analyses should be performed. Second, we generated heatmaps that illustrated the temperature index of all replicas over the course of the simulation. These plots were used to confirm that temperature mixing occurred throughout the replicas, which makes the sampling efficient and provides confidence of good conformational sampling at the temperature of interest (298 K).

Fig. S1A is the potential of mean force (PMF) as a function of radius of gyration for the MOGK2-GpG complex, showing the relative free energy of the complex at different conformational states. The PMF profiles for the 0-300 ns and 0-400 ns intervals show minimal separation compared to the earlier intervals, indicating convergence. Based on this, we used the last 200 ns of the simulation to calculate structural properties of the $MOGK_2$/GpG complex. Next, we analyzed temperature mixing of the $MOGK_2$ simulation (Fig. S1B). The heatmap displays the temperature index of each replica (y-axis) over the course of the simulation (x-axis), where red indicates high temperature and blue indicates low temperature. The substantial mixing of red and blue colors across all replicas and time confirms that efficient temperature mixing occurred throughout the $MOGK_2$ simulation, ensuring thorough conformational sampling. Similar observations were made for $MOGR_2$ (**Fig. S1C-D**), KMOGK (**Fig. S1E-F**), $MOGK_9$ (**Fig. S2A-B**), and $MOGR_9$ (**Fig. S2C-D**).

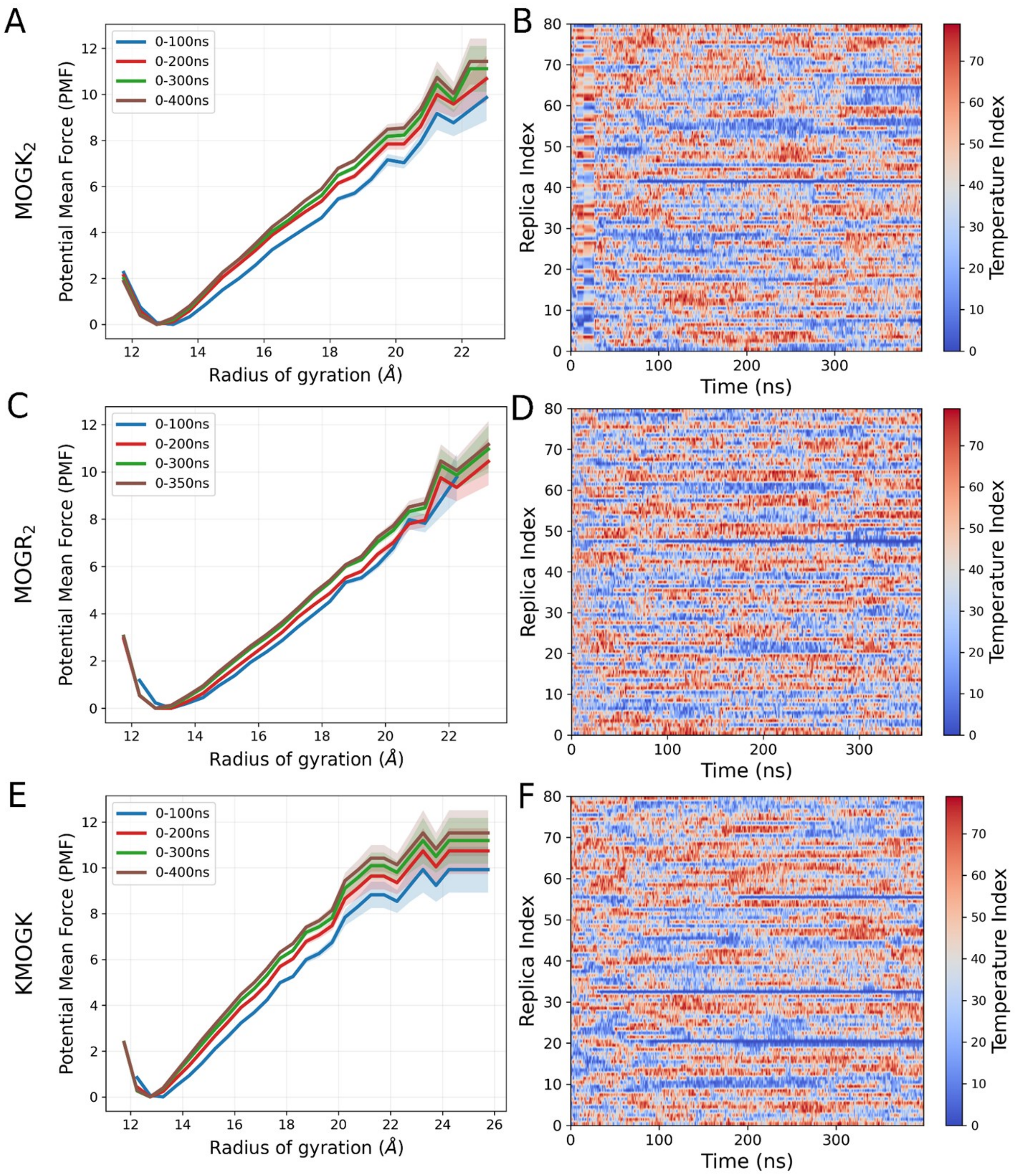

Figure S1: Convergence analysis for replica exchange molecular dynamics simulations of (A-B) MOGK$_2$, (C-D) MOGR$_2$, and (E-F) KMOGK complexes with GpG. (A, C, E) Free energy profiles (PMF in kT units) as a function of radius of gyration calculated over different time intervals during the simulations. Overlapping curves indicate convergence. (B, D, F) Temperature index for each replica over the course of the simulations, demonstrating mixing between different temperature indices. All data were sampled at 298 K.

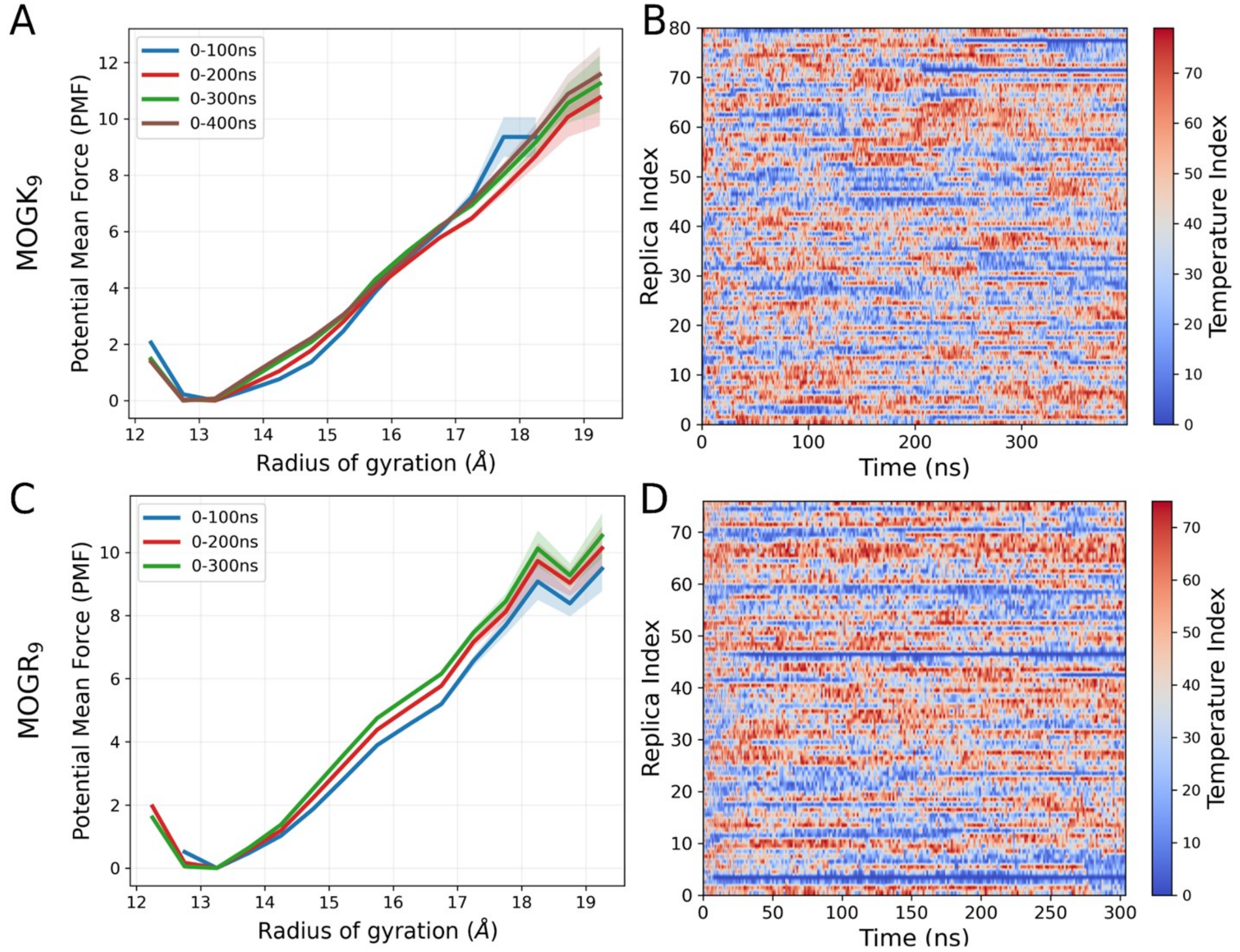

Figure S2: Convergence analysis for replica exchange molecular dynamics simulations of (A-B) $MOGK_9$ and (C-D) $MOGR_9$ complexes with GpG. (A, C) Free energy profiles (PMF in kT units) as a function of radius of gyration calculated over different time intervals during the simulations. Overlapping curves indicate convergence. (B, D) Temperature index for each replica over the course of the simulations, demonstrating mixing between different temperature indices. All data were sampled at 298 K.

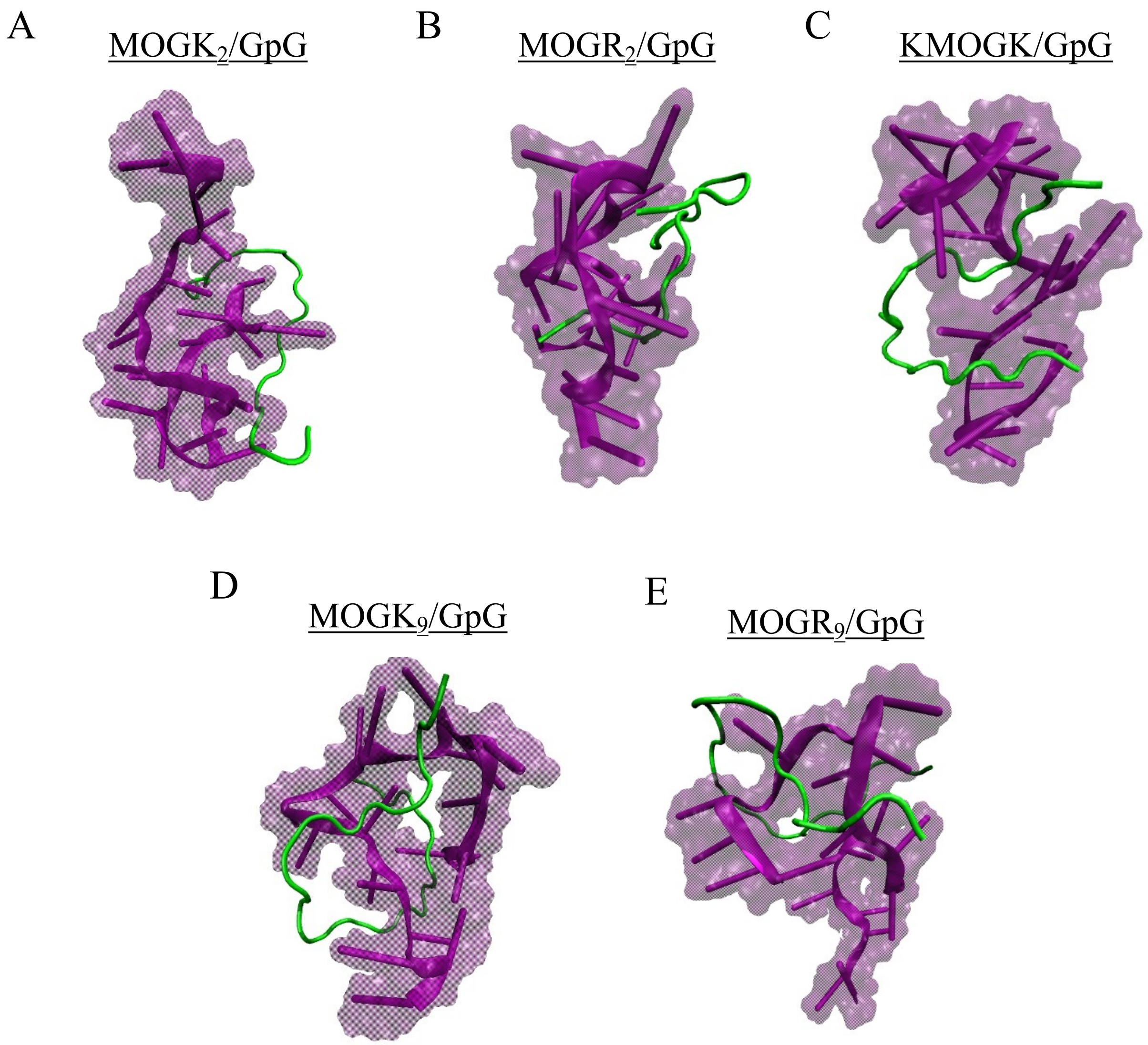

Figure S3: Atomistic renderings of representative self-assembled configurations of (A) $MOGK_2$ and GpG, (B) $MOGR_2$ and GpG, (C) KMOGK and GpG, (D) $MOGK_9$ and GpG, and (D) $MOGR_9$ and GpG.

Materials and Methods

Materials and software. For molecular dynamics simulations, MOG peptide and GpG oligonucleotide structures were generated with The PyMOL Molecular Graphics System, Version 2.0 Schrodinger, LLC, and UCSF Chimera, respectively. Simulations were set up using the CHARMM36m forcefield[1] and executed with the freely available GROMACS software package.[2] All simulations were completed with resources from University of Maryland's High Performance Computing Cluster made available for conducting research reported in this manuscript. Data analysis was completed with both Python and Matlab.

For surface plasmon resonance studies, GpG DNA (5'-T*G*A*C*T*G*T*G*A*A*G*G*T*T*A*G*A*G*A*T*G*A*-3') was purchased from IDT (Coralville, IA). GpG was synthesized with a biotin tag on the 5' end. MOG (MEVGWYRSPFSRVVHLYRNGK) peptides were synthesized by Genscript (Piscataway, NJ). MOG peptides were anchored with either arginine (R) or lysine (K) residues on the C-terminus or both the N-terminus and C-terminus. The MOG peptides studied by surface plasmon resonance included MOG, $MOGK_2$, $MOGR_2$, KMOGK, and RMOGR. HBS-N running buffer (0.01 M HEPES, 0.15 M NaCl, pH 7.4, filtered, degassed), 10 mM sodium acetate pH 5, amine couple kits (1-ethyl-3- (3- dimethylaminopropyl) carbodiimide (EDC) and N-hydroxysuccinimide (NHS)), 1 M ethanolamine, and Series S CM4 sensor chips were provided by Cytiva. Ethylenediaminetetraacetic acid (EDTA) was purchased from Sigma Aldrich (St. Louis, MO). CHARMM36m was selected for its improved treatment of flexible and intrinsically disordered peptide conformational ensembles.[17] It has been benchmarked against experimental observables sensitive to noncovalent interactions, including hydrogen-bond-related NMR metrics and internal dynamics.[18] As CHARMM36m is non-polarizable, we interpret simulation data in terms of relative trends across closely related peptide variants under an identical Hamiltonian rather than absolute binding free energies.

Temperature replica exchange molecular dynamics (TREMD) simulations. The initial individual random structures of MOG peptide and non-phosphorothioated GpG oligonucleotide sequences were generated with Pymol[3] and Chimera, respectively. These individual structures were then assembled to generate MOG/GpG systems for each MOG peptide, with each system containing a single MOG peptide and a single GpG oligonucleotide at center-of-mass separation of 1 nm. A total of 5 systems were created: $MOGK_2$ + GpG, $MOGR_2$ + GpG, $MOGK_9$ + GpG, $MOGR_9$ + GpG, and KMOGK + GpG. The biomolecular systems were then solvated in a $(10\ nm)^3$ periodic cubic box, and 150 mM monovalent ions (Na^+ and Cl^-) were added using CHARMM-GUI solution builder.[1] Then, molecular dynamics (MD) simulations were run with CHARMM36m forcefield[4] and TIP3P[5] water model using GROMACS 2023[6] on the University of Maryland's High Performance Computing Cluster. The simulation protocol followed the standard CHARMM-GUI equilibration protocol and simulation input files. Following initial energy minimization by gradient descent, the systems were equilibrated for 1ns using the NVT ensemble. A 2fs integration time-step was used along with the bonds between the hydrogen atom to the corresponding heavy atom constrained with LINCS[7] and position-restraints on the protein and oligonucleotide. Nose-Hoover thermostat[8] with coupling of 1 ps was used to maintain temperature at 303K. The Lennard-Jones interactions were treated with a Force-switch modifier, switching from 1 nm to the cutoff at 1.2 nm. Finally, the long-range electrostatics was calculated using the Particle-Mesh Ewald (PME) scheme.[9] After equilibration, an initial 50ns simulation with NPT (constant-pressure) ensemble,

without position restraints, was completed for each individual system to allow self-association. In this step, constant pressure was maintained with the Parrinello-Rahman barostat[10] at 1 bar, with a coupling constant of 5 ps. The MOG/GpG conformations found at the end of this step were used as the starting conformations in subsequent simulations. These initial simulations resulted in self-association of the oligonucleotide and the peptide, which allowed using a smaller simulation box for the more computationally intensive Temperature Replica Exchange MD (T-REMD) simulations.

After the initial simulation was completed, water and salt ions were removed from each system, leaving just the complexed MOG peptide and GpG oligonucleotide. Each system was then set up again for T-REMD using the Enhanced Sampler tool on CHARMM-GUI.[11] During this step, the system was solvated and 150 mM ions were added to the simulation box. The simulation setup was prepared with CHARMM36m forcefield, with the TIP3P water model. For the T-REMD options, replicas were distributed between 298 K and 440 K with an exchange probability of 0.21, following the temperature distribution: 298.00, 299.53, 301.07, 302.61, 304.16, 305.72, 307.31, 308.88, 310.45, 312.04, 313.63, 315.22, 316.82, 318.43, 320.05, 321.67, 323.30, 324.94, 326.58, 328.23, 329.90, 331.57, 333.24, 334.92, 336.60, 338.29, 339.99, 341.69, 343.41, 345.13, 346.86, 348.59, 350.33, 352.08, 353.84, 355.60, 357.37, 359.15, 360.94, 362.73, 364.53, 366.34, 368.15, 369.98, 371.81, 373.64, 375.49, 377.34, 379.21, 381.08, 382.96, 384.84, 386.73, 388.63, 390.54, 392.46, 394.39, 396.32, 398.26, 400.21, 402.17, 404.13, 406.11, 408.09, 410.08, 412.08, 414.08, 416.11, 418.13, 420.16, 422.21, 424.26, 426.31, 428.38, 430.46, 432.54, 434.63, 436.74, 438.85, and 440.00 K. The energy minimization and subsequent equilibration of each system followed the exact protocol detailed in the previous paragraph. Finally, the replica-exchange simulations were run in an NVT Ensemble with about 300-400ns per replica, until convergence was achieved.

Computational analysis. We used the in-built functions of MDAnalysis to identify and extract values for radius of gyration, total contacts, hydrogen bonds, and salt bridges.[12] Total contacts were calculated by computing the distance of all non-hydrogen atoms on the MOG peptide and all non-hydrogen atoms on the GpG oligonucleotide. Any non-hydrogen atoms within a 4.5 Å distance were recorded as a contact. Only hydrogen bond pairs between an atom on MOG peptide and an atom on GpG oligonucleotide were computed using a cutoff of 3 Å, and the total hydrogen bonds formed were recorded for each frame. Salt bridges were identified as a contact (4 Å cutoff) between a nitrogen atom on arginine, lysine, or histidine residues of MOG peptide and an oxygen atom on GpG oligonucleotide.[13, 14] The trajectories were sub-sampled to reduce temporal correlation and generate statistically independent samples. Finally, Potential of Mean Force (PMF) and the associated uncertainties for each reaction-coordinate (radius of gyration, total contacts, hydrogen bonds, and salt bridges) was estimated at 298K using the Multistate Bennet Acceptance Ratio (MBAR).[15]

Surface plasmon resonance. Experiments were performed on the Biacore T200 instrument. GpG was immobilized to a Series S CM4 sensor chip and the different MOG peptides were flowed over the chip to analyze binding affinity between MOG and GpG. Flow rates during the experiment were 30 ul/min unless indicated otherwise and detection temperature was 25°C. To start, the CM4 chip was activated with an 8 minute injection of a mixed solution of 0.4 M EDC and 0.1 M NHS. Neutravidin was diluted in 10 mM sodium acetate pH 5 to a final concentration of 50 ug/mL and injected for 10 minutes to couple neutravidin to the sensor chip. The surface was then blocked with a 7 minute injection of 1 M ethanolamine pH 8.5 to remove any remaining reactive species on the

surface. After coupling neutravidin to the surface, biotinylated GpG was immobilized on the surface by diluting GpG in HBS-N running buffer to 1 ug/mL and injecting for 20-60 seconds. To measure kinetic curves, all MOG peptides were diluted in HBS-N running buffer to concentrations of 4800, 3600, 2400, and 1200 nM and flown over the sensor chip with a total contact time of 2 minutes. HBS-N buffer with no peptide (0 nM) was used as a baseline for each run. In between each kinetic curve, the surface of the chip was regenerated with a single injection of HBS-N running buffer for 60 seconds. For each MOG peptide, the five concentrations (including 0 nM) were fitted to a two-state binding model and K_D was calculated based on the association and dissociation rates between MOG peptides and GpG.

***In vitro* polyplex formation.** GpG DNA (5'-TGA CTG TGA AGG TTA GAG ATG A-3') was purchased from IDT (Coralville, IA). MOG_{35-55} (MEVGWYRSPFSRVVHLYRNGK) was synthesized by Genscript (Piscataway, NJ) with arginine (R) or lysine (K) tags on the N-terminus, C-terminus, or both as indicated by the sequence in figures. Polyplexes formed spontaneously upon mixing GpG with modified MOG. The total mass of GpG was fixed at 10µg while the mass of modified MOG was adjusted to achieve a 1:1 charge ratio.

***In vitro* DC Studies**. Splenic DCs were isolated from C57BL/6 mice with CD11c positive magnetic isolation kit (Miltenyi Biotec) following the manufacturer's protocol. After isolation, the cell pellet was resuspended in 1 mL of DC medium (RPMI1640, 10% FBS, 0.5% pen/strep, 50 µM 2-mercaptoethanol (2-ME)) prior to use. 1 x 10^5 DCs in 200µL were plated in each well of a 96 well plate. DCs were activated by adding CpG (TLR9 agonist) at a concentration of 5 µg/mL and treated with each polyplex formulation so that GpG was at a concentration of 1 µg/mL. Controls for this experiment were untreated wells, free CpG only, or free CpG and free GpG. 6 hours after treatment, cells were analyzed for gene expression using RT-qPCR. A separate set of cells were centrifuged for 5 minutes at 300xG to gently pellet cells while leaving polyplexes in solution. Supernatants containing polyplexes were removed, and cells were resuspended in fresh media containing CpG at a concentration of 5 µg/mL. 18 hours af[16]ter this media change, supernatants were collected and analyzed using ELISA assay.

RT-qPCR. For gene expression analysis, RNA was isolated using the Quick-RNA Microprep Kit (Zymo Research, R1050), where cells were lysed in their wells using a lysis buffer, genomic material was captured in a silica-based matrix, and DNA was degraded with DNase I. RNA was diluted to 15ng/µL in RT-qPCR grade water (Thermo Fisher, AM9935). cDNA was reverse transcribed using the High-Capacity cDNA Reverse Transcription Kit (Thermo Fisher, 4368813). The qPCR reaction mix was made using TaqMan Gene Expression Assay probes in TaqMan Gene Expression Master Mix (Thermo Fisher, 4369016).Taqman probes utilized were: glyceraldehyde 3-phosphate dehydrogenase (Gapdh), Mm99999915_g1; actin beta (Actb), Mm00607939_s1; 18s rRNA (18s), Mm03928990_g1; interleukin 1b (Il1b), Mm00434228_m1; interleukin 6 (Il6), Mm00446190_m1; interleukin 12b (Il12b), Mm99999067_m1; interferon gamma (Ifng), Mm01168134_m1; cluster of differentiation 40 (Cd40), Mm00441891_m1; tumor necrosis factor (Tnf), Mm00443258_m1; myeloid differentation primary response 88 (Myd88), Mm00440338_m1 and toll-like receptor nine (Tlr9), Mm00446193_m1. qPCR was performed in a MicroAmp Optical 384-well reaction plate (Applied Biosystems, 4309849) with optical adhesive film (Applied Biosystems, 4360954) on a QuantStudio 7 Flex Real-Time PCR System (Applied

Biosystems, 4485701). Sample normalization was done using an average of three housekeeping genes: Gapdh, Actb and 18s.

IL-6 enzyme-linked immunosorbent assay (ELISA). Supernatants from cultures of splenic DCs with polyplexes were quantified by sandwich ELISA according to the manufacturer's protocols for the secretion of IL-6 (Invitrogen).

Statistical analysis. Statistical analysis for the modeling data was completed during the MBAR analysis. As mentioned, the trajectories of each replica in each simulation were reconstructed to reflect their true temporal correlation, and then subsampled to produce statistically independent samples. Explanations of how uncertainties for free energies and PMF were estimated can be found from the source.[24] For surface plasmon resonance, one-way ANOVA with Tukey post-test corrections for multiple comparisons was used to compare groups. Statistical calculations were performed using JMP Pro (v14, SAS institute). For analysis of treatment impact using RT-qPCR gene expression, data were centered on the negative control. Analysis and hierarchal clustering were done in MATLAB v.R2019b using the clustergram function, where the data were standardized for each gene to compare across multiple groups, and clustering was performed using a single linkage (nearest neighbor). For both gene expression and the IL-6 ELISA assay, data were analyzed by multiple group comparison using one-way analysis of variance (ANOVA) with a Tukey post-test in GraphPad Prism v.6.02. Statistical significance was defined at p values <0.05 (95% confidence interval) and indicated as * = $p \leq 0.05$, ** = $p \leq 0.01$, *** = $p \leq 0.001$, and **** = $p \leq 0.0001$.

References

1. BR, B. et al. CHARMM: the biomolecular simulation program. *J. Comput. Chem.* **30**, 1545-1614 (2009).
2. S, P. et al. GROMACS 4.5: a high-throughput and highly parallel open source molecular simulation toolkit. *Bioinformatics* **29**, 845-854 (2013).
3. Schrödinger LLC The PyMOL Molecular Graphics System, Version 2.0. (2015).
4. Huang, J. et al. CHARMM36m: an improved force field for folded and intrinsically disordered proteins. *Nature Methods* **14**, 71-73 (2017).
5. C, P., F, R. & D, K. On the Choice of Different Water Model in Molecular Dynamics Simulations of Nanopore Transport Phenomena. *Membranes* **12** (2022).
6. Lindahl, A., Hess, & van der Spoel GROMACS 2019.4 Source code (2019.4). *Zenodo* (2019).
6. Mark Abraham, et al. GROMACS 2023 Source code (2023). Zenodo (2023)
7. Hess, B., Bekker, H., Berendsen, H.J.C. & Fraaije, J.G.E.M. LINCS: A linear constraint solver for molecular simulations. *Journal of Computational Chemistry* **18**, 1463-1472 (1997).
8. Evans , D.J. & Holian, B.L. The Nose–Hoover thermostat. *J. Chem. Phys.* **83**, 4069–4074 (1985).
9. Darden, T., York, D. & Pedersen, L. Particle mesh Ewald: An *N*·log(N) method for Ewald sums in large systems. *J. Chem. Phys.* **98**, 10089–10092 (1993).

10. Parrinello, M. & Rahman, A. Polymorphic transitions in single crystals: A new molecular dynamics method. *J. Appl. Physical.* **52**, 7182–7190 (1981).
11. Suh, D. et al. CHARMM-GUI Enhanced Sampler for various collective variables and enhanced sampling methods. *Protein science : a publication of the Protein Society* **31** (2022).
12. N, M.-A., EJ, D., TB, W. & O, B. MDAnalysis: a toolkit for the analysis of molecular dynamics simulations. *J. Comput. Chem.* **32**, 2319-2327 (2011).
13. Kumar, S. & Nussinov, R. Close-range electrostatic interactions in proteins. *Chembiochem.* **3**, 604-617 (2002).
14. Donald, J.E., Kulp, D.W. & DeGrado, W.F. Salt bridges: geometrically specific, designable interactions. *Proteins* **79**, 898-915 (2011).
15. Shirts, M.R. & Chodera, J.D. Statistically optimal analysis of samples from multiple equilibrium states. *J. Chem. Phys.* **129**, 124105 (2008).
16. Lindahl, A., Hess, & van der Spoel GROMACS 2019.4 Source code (2019.4). (2019).
17. Huang, J. et al. CHARMM36m: an improved force field for folded and intrinsically disordered proteins. *Nature Methods*. **14**, 71-73 (2017).
18. Xu, Y., Huang, J. Validating the CHARMM36m protein force field with LJ-PME reveals altered hydrogen bonding dynamics under elevated pressures. *Communications Chemistry*. **4**, 99 (2021).
